# Effect of NLRP3 inflammasome genes polymorphism on disease susceptibility and response to TNF-α inhibitors in Iraqi patients with rheumatoid arthritis

**DOI:** 10.1016/j.heliyon.2023.e16814

**Published:** 2023-06-01

**Authors:** Abdullah Abbas Awni, Zainab Oday Hamed, Ahmed Abdul-Hassan Abbas, Ahmed Sahib Abdulamir

**Affiliations:** a College of Medical Sciences Techniques, The University of Mashreq, Baghdad, Iraq; b Therapeutics and Clinical Pharmacy Department, Baghdad College of Medical Sciences, Baghdad, Iraq; c Microbiology Department, College of Medicine, Al-Nahrain University, Baghdad, Iraq

**Keywords:** Rheumatoid arthritis, Etanercept, Infliximab, IL-1β, TNF-α, NLRP3, CARD8, Single nucleotide polymorphism (SNP)

## Abstract

**Background:**

Rheumatoid arthritis (RA) is a genetically predisposed, systemic, chronic, inflammatory disease. Immune system dysregulation and inherited susceptibility polymorphisms suggest that this type of variation is functional and may help predict disease susceptibility and develop new therapeutic strategies. Anti-TNF-alpha (TNF-α) drugs are highly effective RA treatments, but not all patients respond the same way. It's important to figure out whether RA risk alleles can identify and predict anti-TNF-α-responsiveness in RA patients.

**Aims of the study:**

Examine the function of the NLR family pyrin domain containing 3 (NLRP3) and caspase recruitment domain family member 8 (CARD8) genes polymorphisms and their morbid genotypes and alleles in RA patients and apparently healthy controls. In addition, their role in disease susceptibility, severity, and response to anti-TNF-α therapy. Also, examine how single nucleotide polymorphisms (SNPs) affect serum levels of pro-inflammatory cytokines like TNF-α and interleukin (IL)-1β.

**Materials and methods:**

100 RA patients (88 females, 12 males) and 100 apparently healthy people (86 females, 14 males) were examined. To measure serum TNF-α and IL-1β, Elabscience sandwich ELISA kits were used. Iraq Biotech, Turkey DNA extraction kit was used to extract genomic DNA from whole blood. CARD8 (rs2043211) and NLRP3 (rs4612666) were genotyped using Agilent, AriaMx, USA, through Tri-Plex SYBR Green-based real-time PCR allelic discrimination assays. Geneious software, version 2019.2.2, used to design primers from published sequences (GenBank accession no. GCA 009914755.1). Primer specificity was determined by NCBI's BLAST.

**Results:**

Study found that there is association between cytokines serum level and 28-joints disease activity score (DAS-28). The level of TNF-α increases with the higher DAS-28 (r^2^ = 0.45, P < 0.0001). Also, IL- 1β level increases with higher DAS-28 (r^2^ = 0.51, P < 0.0001). There were no statistically significant variations between patients with RA and the control group in the distribution of CARD8 SNP rs2043211 and NLRP3 SNP rs4612666 genotypes (P = 0.17 and 0.08 respectively) as well their alleles (P = 0.059 and 0.879 respectively). CARD8 (rs2043211) TT genotype was more frequent in patients with higher DAS-28 (P < 0.0001) and higher TNF-α and IL-1β serum levels (P < 0.0001 for both). Also, NLRP3 (rs4612666) TT genotype was more frequent in patients with higher DAS-28 (P < 0.0001) and higher TNF-α and IL- 1β serum levels (P < 0.0001 for both). Interestingly, this study revealed that CARD8 (rs2043211) and NLRP3 (rs4612666) variant genotypes are associated with lower response to anti-TNF-α drugs.

**Conclusions:**

Serum TNF-α and IL-1β correlate with DAS-28 and disease activity. Non-responders have elevated TNF-α and IL-1β. CARD8 rs2043211 and NLRP3 rs4612666 variant polymorphisms are associated with high serum TNF-α and IL-1β, active disease course, poor disease outcomes, and low response to anti-TNF-α therapy.

## 1 Introduction

Rheumatoid arthritis (RA) is an autoimmune disease characterized by chronic inflammation and joint damage [1]. Genetic and environmental factors have been implicated in RA pathogenesis, but the exact causes are unknown. RA pathogenesis also involves abnormal immune responses, both innate and adaptive. Many cytokines are involved in RA pathogenesis and persistence [2]. As producers of cytokines and antigen presentation by phagocytes, innate immune system cells are crucial to disease initiation and maintenance [3]. Monocytes/macrophages that release tumor necrosis factor (TNF)-α and interleukin (IL)-1β contribute to joint inflammation. B-lymphocytes (B-cells) could control the immune response direction through antibody production by independent mechanism. Such processes include the presentation of antigen and the release of pro-inflammatory cytokines [4].

Innate and adaptive immune system activation are linked. Several studies have shown that NLR family pyrin domain containing 3 (NLRP3) inflammasome affects this relationship [5]. NLRP3, a complex multimeric protein, controls inflammatory immune response, apoptosis, and the release of pro-inflammatory cytokines like IL-1β as an upstream activator of nuclear factor κ-light-chain-enhancer of activated B-lymphocytes (NF-κB) signaling [6]. Inflammasome activation is tightly regulated by many processes, including transcription of NF-κB-dependent NLRP3 and IL-1β and endogenous proteins like caspase domain-containing protein 8 (CARD8), which physically interacts with caspase-1 and negatively regulates caspase-1-dependent IL-1β expression and NF-κB activation [7].

CARD8 and NLRP3 polymorphisms affect RA susceptibility and severity, according to several studies. Addobbati et al. (2018) found that CARD8 rs2043211 SNP (A > T) is associated with disease severity in RA clinical manifestations [8]. Patients homozygous for the T allele had higher functional disability. The rs2043211 polymorphism introduces a premature stop codon (Cysteine > Stop) that produces a severely truncated protein [9]. CARD8 role in inflammasome biology is unknown. CARD8 may modulate NLRP3 activation or induce NF-κB inflammasomes independently [10]. CARD8 variation increases IL-1β secretion, especially when combined with NLRP3 rs35829419 [11]. In addition, this variation associated with increased induction and translocation of NF-κB activity to the nucleus, resulting in high constitutive levels of RA inflammatory mediators like pro-IL-1β and TNF-α [12]. Synovial cell proliferation and bone and cartilage degradation have also been linked to NF-κB [13].

RA treatment aims to promote remission or very low disease activity. Biological therapies improve clinical and radiographic outcomes for some RA patients. Hypoxia activates the NLRP3-inflammasome in active RA. Therefore, it is hypothesized that NLRP3-inflammasome is activated before biologics in RA patients and that anti-TNF-α therapy may modulate this activation [14].

NLRP3 gene polymorphism rs4612666T was associated with decreased expression. There was a relationship between two variants of NLRP3 (rs4925659 and rs10925026) and the European League Against Rheumatism (EULAR) response. rs10925026 and rs4925648 in NLRP3 were associated with good versus no response. Rs10925026 C allele was associated with non-response, while rs4925659's A allele with improved response. rs4925659 variant had modest RA susceptibility with (rs10159239), but not rs10925026 or rs4925648 [14]. Kastbom et al. (2010) found a link between CARD8 p. C10X polymorphism and RA severity in Swedish and Spanish patients. (c.30 TA, rs2043211) produces a premature stop codon (p.C10X). Compared to NLRP3/CARD8+/+, the combination of p. Q705K/p. C10X in NLRP3/CARD8 (−/−, denoting the two minor alleles) was significantly more frequent in RA patients [15]. In early RA studies, the p. C10X CARD8 variant was linked to inflammatory activity and overrepresented in anti-TNF-α patients. RA has persistent innate immune system activation [16].

European Alliance of Associations for Rheumatology (EULAR) response was nominally correlated with CARD8 SNP (rs11672725), with the T allele enhancing response [8].

Disease susceptibility and response to anti-TNF-α drugs suggest different genetic regulatory mechanisms in RA patients [17]. Anti-TNF-α agents have greatly improved RA symptoms, but not all patients respond [18]. Better diagnosis, treatment, and healthcare cost reduction are new concerns [19]. The discovery of inherited variations (polymorphisms) and their relationship to immune system dysregulation, immune complex-mediated disease, and disease susceptibility suggests that this type of variation is functional and evolutionary and can help predict disease susceptibility and develop new therapeutic strategies [20]. It is essential to determine whether these RA risk genotypes or alleles can pinpoint RA predisposition and predict patients’ response to biological drug before therapy. NLRP3 SNP rs4612666 and CARD8 SNP rs2043211 were examined in RA patients and healthy controls to determine their morbid genotypes and alleles. Additionally, examine the association of gene polymorphisms with disease susceptibility, severity, response to anti-TNF-α therapy, and serum TNF-α and IL-1β levels.

## 2 Patients, materials and methods

The study recruited 100 patients with EULAR-defined RA and 100 apparently healthy subjects. All signed a biomedical research consent form. Ethical approval was obtained from the Institutional Review Board (IRB) - College of Medicine/Al-Nahrain University. All patients were diagnosed and treated by rheumatologists at Baghdad Teaching Hospital - Rheumatology Unit from November 2018 to March 2020. RA patients with other chronic diseases and those treated with Etanercept or infliximab for less than 3 months were excluded from the study. As a control, apparently healthy subjects of similar age and sex were recruited. Venous blood was collected for genetic studies and measurement of serum level TNF-α and IL-1β. Age, gender, number of tender (TEN) and swollen (SW) joints were collected from RA patients. Visual analogue scale (VAS) of pain and disease activity were assessed by rheumatologist, Also, type of anti-TNF-α drug. DAS-28 assessed clinical disease activity, including remission, according to EULAR criteria. DAS-28 scores were used to divide patients into responders and non-responders subgroups (Table 1).

**Table 1:** EULAR-defined disease activity and treatment response after 3 months.

DAS-28 score	Disease activity/Response
DAS-28 < 2.60	Remission
DAS-28 ≥ 2.60 and ≤ 3.20	Low or mild disease activity
DAS-28 > 3.20 and ≤ 5.10	Moderate disease activity
DAS-28 > 5.10	High or severe disease activity
ΔDAS-28 ≥ 1.2 and DAS-28 ≤ 3.2	Responders
ΔDAS-28 ≤ 1.2 and DAS28 ≤ 5.1	Non-responders

Genomic DNA was extracted from EDTA-anti-coagulated venous blood using Iraq Biotech Co., Turkey DNA extraction kit from whole blood according to the manufacturer's instructions. Primers were designed according to the published sequences (GenBank accession no. GCA_009914755.1) using Geneious software, version 2019.2.2. The software introduced a mismatch at the −2 position of the 3′ end of both allele-specific primers to increase reaction specificity (Table 2). The lyophilized primers were prepared according to Integrated DNA Technologies' (IDT, USA) instructions. BLAST program at national center for biotechnology information (NCBI) was used to determine the specificity of the primers (www.ncbi.nlm.nih.gov/blast).

**Table 2:** The NLRP3 rs4612666 and CARD8 rs2043211 SNP primer sets.

Gene	SNP	Primers	Set Ta
CARD8	rs2043211-COM	TTTCTGAGACCCTTTGTGACAT	54.7
	rs2043211-A	CAGGAACAGCACGGAACA	
	rs2043211-T	CAGGAACAGCACGGATCA	
NLRP3	rs4612666-COM	ACAACAATGTGAAGTGCTTA	51.2
	rs4612666-A	GCTCCCACCAATACTACA	
	rs4612666-G	GCTCCCACCAATACTACG	

Ta: annealing temperature.

CARD8 (rs2043211) and NLRP3 (rs4612666) were genotyped using SYBR Green-based Tri-Plex real-time PCR allelic discrimination assay using AriaMx, Agilent, USA. In a total volume of 20 μL containing 10 μL of 2x SYBR Green PCR Master, 1 μL of each primer per reaction, 2 μL of the genomic DNA dilution, and distilled water to the final volume was mixed. The PCR thermal profile was as follows: an initial denaturation step (95 °C for 3 min, 1 cycle) was followed by amplification and quantification steps repeated for 40 cycles (95 °C for 5 s, primer set annealing temperature for 15sec (54.7 for CARD8 SNP and 51.2 for NLRP3 SNP), 72 °C for 20 s, with a single fluorescence measurement at the end of the elongation step at 72 °C). A high-resolution melting curves (HRMs) were constructed by lowering the temperature to 55 °C and later increasing the temperature by 0.2 °C each 30 s to 95 °C while continuously measuring the change in fluorescence. Melting temperature (Tm) values were automatically assigned a plot generated by the Agilent AriaMx software. Sandwich-ELISA Kits from Elabscience, USA, was used to measure TNF-α and IL-1β serum levels. The procedure was carried out in accordance with the manufacturer instructions. BioTek ELISA reader, USA, was used for all measurements. Statistical analysis operated using SPSS Statistics (V 25.0; IBM SPSS Software, USA). A P < 0.05 (exact two-sided) was accepted as level of significance. Continuous variables were subjected to a normality test. If the data had a normal distribution, the *t*-test or analysis of variance (ANOVA) was used to compare means. Therefore, these variables were expressed as mean ± standard deviation (SD). Binomial variables were expressed as numbers and percentages and analyzed with Chi-square test. Binary logistic regression was used to calculate odds ratios (ORs) and the corresponding 95% confidence intervals (CIs) in order to assess the association between polymorphisms different genotypes and alleles in RA patients.

## 3 Results

### 3.1 Demographic and clinical features of the patients and controls

The proportion of females to males in both the patient and control groups were comparable statistically (P = 0.67). RA is 7.33 times more common in women than men, females made up 88% of RA patients.

The mean age of female patients was 49.84 (±11.69) and the mean age of female controls was 53.09 (±10.52), P = 0.056. The mean age of males in patients and controls was 39.33 (±9.59) and 44.86 (±10.26), respectively, P = 0.171 (Table 3).

**Table 3:** Sex and age of patients and control participants.

Parameters		Patient (n = 100)	Control (n = 100)	Total (n = 200)	P value
Sex Count (Percent)	Female	88 (88%)	86 (86%)	174 (174%)	0.674
	Male	12 (12%)	14 (14%)	26 (26%)	
Age (Years) Mean (±SD)	Female	49.84 (11.69)	53.09 (10.52)	51.45 (11.22)	0.056
	Male	39.33 (9.59)	44.86 (10.26)	42.31 (10.15)	0.171

Thirty-four of 100 RA patients (14 etanercept and 20 infliximab) did not respond to anti-TNF-α therapy, while 66 (38 and 28) did. Table 4 shows that the difference in treatment response between the two drugs was not statistically significant (P = 0.120). Moreover, after 3 months of treatment, the DAS-28 was 3.89 (±0.98) compared to 4.92 (±1.29) at baseline. Etanercept and infliximab had similar DAS-28 scores after 3 months (P = 0.88). 63% of RA patients had moderate disease activity, 19% low, and 10% high. Only 8% of patients had remission after 3 months of treatment. As shown in Table 4, the effects of the two drugs were comparable (P = 0.32). Most RA males responded better to anti-TNF-α than females (P = 0.045) and showed lower DAS-28 after 3 months, with mean of 2.77 versus 4.04 respectively (P = 0.0001), despite baseline mean was not significantly different between sexes (P = 0.902). The disease activity was significantly different between males and females (P < 0.0001), and males were more likely to respond to anti-TNF-α treatment after 3 months, showing remission or low disease activity than females. High disease activity was found in 0/12 male patients.

**Table 4:** Clinical characteristics of RA patients.

Parameters		Type of Drug			P value	Sex			P value
		Etanercept (n = 52)	Infliximab (n = 48)	Total		Female (n = 88)	Male (n = 12)	Total	
Response Count (Percent)	Non-responders	14 (14%)	20 (20%)	34 (34%)	0.120	33 (33%)	1 (1%)	34 (34%)	0.045
	Responders	38 (38%)	28 (28%)	66 (66%)		55 (55%)	11 (11%)	66 (66%)	
DAS-28 score Mean (±SD)	Baseline	5.30 (1.08)	4.51 (1.39)	4.92 (1.29)	0.002	4.92 (1.27)	4.97 (1.52)	4.95 (1.29)	0.902
	After 3 months	3.90 (0.91)	3.87 (1.07)	3.89 (0.98)	0.881	4.04 (0.91)	2.77 (0.76)	3.89 (0.98)	<0.0001
Disease activity after 3 months Count (Percent)	Remission	2 (2%)	6 (6%)	8 (8%)	0.321	4 (4%)	4 (4%)	8 (8%)	<0.0001
	Low	11 (11%)	8 (8%)	19 (19%)		13 (13%)	6 (6%)	19 (19%)	
	Moderate	35 (35%)	28 (28%)	63 (63%)		61 (61%)	2 (2%)	63 (63%)	
	High	4 (4%)	6 (6%)	10 (10%)		10 (10%)	0 (0%)	10 (10%)	

RA patients had higher TNF-α levels than controls, with a mean concentration of 7.84 pg/mL versus 6.09 pg/mL, P = 0.0005. IL-1β levels were also significantly higher in patients than controls, with a mean concentration of 2.18 pg/mL versus 1.21 pg/mL respectively (P < 0.0001). In RA patients taking infliximab or etanercept, serum TNF-α and IL-1β levels were measured. Etanercept and infliximab-treated RA patients had similar IL-1β levels (P = 0.168). TNF-α was higher in infliximab-treated patients, but not significant (P = 0.088). DAS-28 correlated positively with TNF-α (r^2^ = 0.45, P < 0.0001) and IL- 1β (r^2^ = 0.51, P < 0.0001). TNF-α and IL-1β concentrations were significantly higher in patients with moderate to high disease activity and lower in those in remission or low disease activity (P = 0.001 and < 0.0001). Most responders had lower IL-1β and TNF-α (P < 0.0001). Low levels of pro-inflammatory cytokines were linked to biologic anti-TNFα agent responsiveness by blood cytokine profiling (Table 5).

**Table 5:** Serum TNF-α and IL- 1β in the patients and control groups.

Parameters		TNF-α		IL- 1β	
		Mean (SD)	P value	Mean (SD)	P value
Group Mean (±SD)	Patients	7.84 (5.57)	0.005	2.18 (1.21)	<0.0001
	Control	6.09 (2.79)		1.20 (0.58)	
Type of drug Mean (±SD)	Etanercept	6.93 (4.67)	0.088	1.97 (1.16)	0.102
	Infliximab	8.83 (6.30)		2.39 (1.38)	
Disease activity after 3 months Mean (±SD)	Remission	4.52 (1.83)	0.001	1.28 (0.48)	<0.0001
	Low	5.34 (2.26)		1.53 (0.61)	
	Moderate	8.15 (6.1)		2.25 (1.3)	
	High	13.31 (3.79)		3.68 (1.22)	
Response Mean (±SD)	Non-responder	13.68 (5.50)	<0.0001	3.36 (1.24)	<0.0001
	Responder	4.83 (2.18)		1.57 (0.58)	
DAS-28 score	(Pearson correlation)	0.45	<0.0001	0.51	<0.0001

### 3.2 Genotype and allele frequencies of CARD8 rs2043211 and NLRP3 rs4612666 polymorphisms in RA

RA patients and controls had similar CARD8 rs2043211 and NLRP3 rs4612666 genotype distributions (P = 0.17 and 0.08 respectively), where no deviations from Hardy–Weinberg equilibrium were detected for the genotype distribution. In addition, patients and controls had comparable CARD8 (rs2043211) and NLRP3 allele frequencies, P = 0.059 and 0.879, respectively (Table 6).

**Table 6:** Genotype and allele frequencies in patients and control groups.

Parameters		CARD8 rs2043211 (A > T)		P value	NLRP3 rs4612666 (C > T)		P value
		Patients	Control		Patients	Control	
Genotype frequencies	Homozygous wild	37	48	0.17	47	42	0.08
	Heterozygous	47	43		42	54	
	Homozygous variant	16	9		11	4	
Allele frequencies		121/79	139/61	0.059	68/32	69/31	0.879
Minor allele frequencies (MAF)		0.395	0.305		0.320	0.310	

rs#: reference SNP number.

### 3.3 Genotype-phenotype analysis of RA patients

The CARD8 (rs2043211) TT genotype was more common in patients with higher DAS-28 (P < 0.0001). Also, DAS-28 was higher in NLRP3 (rs4612666) TT genotypes (P < 0.0001). CARD8 (rs2043211) genotypes distribution in patients showed a significant difference in disease activity (P = 0.033). There are also significant differences in NLRP3 (rs4612666) genotype distribution (P = 0.008).

Significant difference was observed in CARD8 (rs2043211) genotypes between responders and non-responders (P < 0.0001). As well as, NLRP3 genotypes (rs4612666) (P < 0.0001).

Comparison of SNPs genotypes with pro-inflammatory cytokines. A significant difference in the CARD8 (rs2043211) genotypes (P < 0.0001 for TNF-α and IL- 1β) as well as, NLRP3 (rs4612666) genotypes (P < 0.0001 for TNF-α and IL- 1β), Table 7.

**Table 7:** Clinical features and serum cytokines of patients with RA and different genotypes of CARD8 rs2043211 and NLRP3 rs4612666 polymorphisms.

Parameters		CARD8 rs2043211 Genotypes				NLRP3 rs4612666 Genotypes			
		AA	AT	TT	p-value	CC	CT	TT	p-value
DAS-28 Mean (SD)		3.51 (0.90)	3.92 (1.00)	4.65 (0.65)	<0.0001	3.52 (0.89)	4.08 (1.00)	4.72 (0.55)	<0.0001
Frequency of Disease activity (n=100)	Remission	5	3	0	0.033	7	1	0	0.008
	Low	9	10	0		7	1	0	
	Moderate	23	27	13		29	25	9	
	High	0	7	3		0	8	2	
Response after 3 months	Responders	32	33	1	0.0001	41	24	1	0.0001
	Non-responders	5	14	15		6	18	10	
Cytokines Mean (SD)	TNF-α	5.64 (3.56)	7.39 (5.59)	14.25 (4.67)	<0.0001	5.56 (3.31)	8.58 (6.17)	14.74 (4.61)	<0.0001
	IL- 1β	1.05 (0.34)	2.25 (0.57)	4.55 (0.64)		1.18 (0.39)	2.59 (0.69)	4.86 (0.50)	

The ANOVA test was performed to determine if the relationship of DAS-28 and SNPs was linked to dominant or recessive models (Table 8). Patients with CARD8 (rs2043211) A allele bearing genotypes (wild homozygous and heterozygous genotypes) associated with lower DAS- as compared to those with T allele bearing genotypes (Variant homozygous genotypes and heterozygous genotypes. This assume that CARD8 (rs2043211) variant genotypes are associated with lower response to anti-TNF-α treatments in RA patients. Moreover, NLRP3 (rs4612666), C Allele bearing genotypes (wild homozygous and heterozygous genotypes) are associated with lower DAS-28 as compared with T allele bearing genotypes (Variant homozygous and heterozygous genotypes (P < 0.0001 and 0.003 respectively), this supposing that NLRP3 (rs4612666) variant genotypes are extremely associated with higher disease progression and lower response to anti-TNF-α drugs.

**Table 8:** Clinical features and serum cytokines of patients with RA and different genetic models of CARD8 rs2043211 and NLRP3 rs4612666 polymorphisms.

Parameters		CARD8 rs2043211 Genetic models				NLRP3 rs4612666 Genetic models			
		Dominant (AA + AT/TT)	p-value/OR (95% CI)	Recessive (AA/AT + TT)	p-value/OR (95% CI)	Dominant (CC + CT/TT)	p-value/OR (95% CI)	Recessive (CC/CT + TT)	p-value/OR (95% CI)
DAS-28 Mean (SD)		3.74 (0.97)/4.65 (0.65)	0.001	3.51 (0.9)/4.1 (0.97)	0.003	3.78 (0.98)/4.72 (0.55)	0.003	3.52 (0.89)/4.21 (0.96)	<0.0001
Disease activity frequency (n=100)	Remission	8/0	0.055	5/3	0.029	8/0	0.182	7/1	0.002
	Low	19/0		9/10		19/0		11/8	
	Moderate	50/13		23/40		54/9		29/34	
	High	7/3		0/10		8/2		0/10	
Response after 3 months	Responders	19/15	<0.0001/0.019 (0.002–0.157)	5/29	0.001/0.183 (0.063–0.531)	24/10	<0.0001/0.037 (0.004–0.304)	6/28	<0.0001/0.131 (0.047–0.36)
	Non-responders	65/1		32/34		65/1		41/25	
Cytokines Mean (SD)	TNF-α	6.62 (4.85)/14.25 (4.67)	<0.0001	6.99 (5.07)/14.74 (4.61)	<0.0001	7.75 (5.79)/8.16 (4.81)	0.764	5.64 (3.56)/9.13 (6.12)	0.002
	IL- 1β	1.72 (0.77)/4.55 (0.64)	<0.0001	1.84 (0.9)/4.86 (0.5)	<0.0001	2.27 (1.38)/1.86 (0.8)	0.187	1.05 (0.34)/2.84 (1.17)	<0.0001

Comparison of SNPs genetic models with disease activity, reveal that higher disease activity is associated with patients harboring CARD8 recessive genetic models (P = 0.029), wherein, higher disease activity was associated with heterozygous and variant homozygous genotypes (T allele bearing) of CARD8 SNP rs2043211 as compared with wild (AA) genotype. Additionally, NLRP3 (rs4612666) recessive model is associated with high disease activity (P = 0.002), similarly, higher disease activity was associated with NLRP3 (rs4612666) heterozygous and variant homozygous genotypes (T allele bearing) as compared with wild (CC) genotype.

Higher response rate associated with dominant models of CARD8 SNP rs2043211 and NLRP3 SNP rs4612666 (P < 0.0001 and 0.001 respectively). While highly non response rate related with recessive models of these two SNPs (P < 0.0001 for both).

ANOVA test was carried out to found out if the association between cytokines serum level and SNPs dominant or recessive model (Table 8). Comparing SNPs models reveal that lower TNF-α and IL- 1β serum level after 3 months of treatment were associated with dominant models of CARD8 (rs2043211) SNPs (P < 0.0001 for both), while higher cytokines level associated with recessive model (P < 0.0001 for both). Recessive model of NLRP3 (rs4612666) was associated with higher TNF-α and IL- 1β serum level after 3 months of treatment (P = 0.002 and P < 0.0001 respectively), where T allele bearing genotypes (Variant homozygous and heterozygous) were significantly associated with higher cytokines level. In contrast, dominant model was not significantly correlated with TNF-α and IL- 1β serum level (P = 0.764 and 0.187 respectively), Table 8.

## 4 Discussion

Tumor necrosis factor-α antagonists have been the most effective tool for controlling patients suffering from a variety of rheumatic diseases over the last two decades. Infliximab and etanercept can induce remission, decrease the disease activity and prevent the progression of both clinical and radiological disease in RA, with a significant improvement in the symptoms, function and quality of life of patients [21]. Approximately one-third of patients display a lack of response to anti-TNF-α therapies, either due to lack of drug efficacy or following the occurrence of adverse events (AEs). There are variations in their site of action and molecular structure, although the 2 drugs exert their beneficial effects by blockade of TNF-α. These differences may explain the differential response to the 2 agents in individual patients, even though there is no direct evidence to support this [22]. A total of 10 studies involving 1806 subjects were included in meta-analysis review, which suggested that anti-TNF-α inhibitor antibodies had a significant association with decreased anti-TNF-α response. Anti-TNF-α agents for RA treatment are unsuccessful in some patients [23]. One causative factor for non-response to anti-TNF-α drugs, such as infliximab and etanercept for RA treatment, is anti-TNF-α inhibitor antibodies produced in patients [24]. Several studies suggest that the main reasons for different clinical responses could be different bioavailability and diverse expression of anti-TNF-α inhibitor antibodies in RA patients [25,26]. The key cause of treatment failure is known to be immunogenicity of the anti-TNF-α agents [27]. Due to the variety of factors such as route of administration, drug dosage, concomitant medication, comprehensive treatment plan, and immune and nutritional status, the immunogenicity rate of different anti-TNF-α drugs is different [28]. Extensive studies have investigated the impact on drug efficacy and safety of the immunogenicity of anti-TNF-α agents and have found that antibodies to these agents, such as infliximab, can affect the pharmacokinetics of the drug, resulting in delayed infusion and injection site reactions [28,29]. Meanwhile, infliximab-induced anti-chimeric antibodies (ATIs) could enhance the clearance of infliximab and attenuate its function [30]. This may be a plausible explanation for the essential association of anti-TNF-α inhibitor antibodies with a reduced response rate.

Current study compared the levels of cytokines in infliximab and etanercept groups in patients with RA who received either treatment. The findings showed that IL-1β and TNF-α levels appeared to be identical in both groups, as both drugs seem to have relatively similar anti-inflammatory properties. These two drugs are both monoclonal antibodies and share a relatively similar mechanism to inhibit production of TNF-α by neutralizing the biological activity of TNF-α by binding to the soluble and transmembrane forms of TNF-α with a high affinity and by inhibiting or preventing the successful binding of TNF-α with its receptors [31]. The only difference is their nature; infliximab is a monoclonal chimeric anti-TNF-α antibody that binds both monomeric and trimeric soluble TNF and transmembrane TNF, with murine variable regions and human IgG1 constant regions. Etanercept is a dimeric fusion protein connected to the Fc domains of human IgG1 consisting of the extracellular portion of TNFRp75. Etanercept binds only trimeric TNF and, compared to infliximab, interacts with transmembrane TNF with reduced avidity [32].

Analysis has identified variations in cytokine levels between RA patients and control group. Mean serum TNF-α levels were significantly higher in RA patients after 3 months of treatment, and IL-1β levels were also significantly higher in the patients group.

There was a significant relation between serum level of cytokines (TNF-α and IL-1β) and disease activity and response to anti-TNF-α agents after 3 months of treatment, where it was directly proportional with it. Cytokines with different levels of expression have also been found in those patients who responded to anti-TNF-α therapy relative to patients who did not. Cytokines can be useful biomarkers of the tendency of a patient to respond to a particular biological therapy.

The pivotal role played by TNF-α and IL-1β in the development and maintenance of inflammation in non-responder RA patients is supported by decreased cytokines in responding patients receiving anti-TNF-α drugs. Another interesting finding is that patients who did not respond (3 months post-therapy) to anti-TNF-α therapy had significantly higher serum IL-1β concentrations. This finding confirms recently published data that showed elevated expression of the IL-1β gene in synovial biopsies from adalimumab therapy non-responders [33]. In inflammatory diseases, IL-1β is an emerging target for drug therapy, and anakinra (Recombinant IL-1 receptor antagonist) has been developed to block IL-1β signaling as a biological therapy [34]. However, indirect associations with adalimumab, etanercept and infliximab have shown a trend towards greater anti-TNF-α drug efficacy [21].

After an insult, TNF-α (and other cytokines) are developed by the cells of the innate immune system, raising and prolonging the inflammatory response by triggering the release of several cytokines by other cells (i.e., IL-1β). Via decreased FOXP3 expression, TNF-α has been shown to suppress Treg function, an effect that can be reversed by TNF-α blockade [35]. Therefore, the elevation of IL-1β and TNF-α in a non-responder serum can be a biomarker of the response to anti-TNF-α therapy. Prospective research will explain if it could be possible to use the ratio of these cytokines to predict response to anti-TNF-α medication. Such a study should obviously be structured to account for possible influencing factors in the diagnosis, such as prior and concurrent drug treatment, duration of the disease.

The goal of the study was to explore the impact of genetic factors on the susceptibility of RA. Selected SNP in the study CARD8 rs2043211 (A > T) and NLRP3 rs4612666 (T > C) have identified the significance of their role in RA pathogenesis in several previous studies. Our results indicate that the 2 SNPs were not associated with RA susceptibility.

The influence of CARD8 rs2043211 and NLRP3 rs4612666 polymorphisms was stated after 3 months of RA treatment. T allele bearing genotypes (Variant homozygous genotypes and heterozygous genotypes) for CARD8 rs2043211 NLRP3 rs4612666 polymorphism had significantly higher DAS-28. Consequently, variant genotypes of CARD8 rs2043211 and NLRP3 rs4612666 were related with higher disease activity and lower response to TNF-inhibitors, also higher concentration of TNF-α and IL-1β.

This may be due to the NLRP3 gain-of-function polymorphism, leading to an overactive NLRP3 inflammasome [35,36], and possibly to increased activation of IL-1β [37]. DAS-28 was highly affected by homozygosity for the polymorphic CARD8 rs2043211 T allele. The findings were consistent with the study documenting the effect of CARD8 rs2043211 on the more serious course of long-lasting RA disease [38].

Truncated CARD8 resulting from CARD8 rs2043211 could decrease its influence over the signaling pathway of NF-κB and thus lead to a more active transcriptional factor and inflammatory process amplification [38]. For the expression of both inflammatory cytokines and tissue destructive enzymes in RA, NF-κB is essential [39,40].

In the gene expression analysis, the contribution of NLRP3 and CARD8 to active RA was shown, in particular in patients with RA, NLRP3 was upregulated and CARD8 was downregulated in contrast to healthy controls. In a well-powered study of UK RA patients, the results verified the association between the NLRP3 gene and the EULAR anti-TNF-α response [14]. Therefore, good and moderate EULAR response versus no response in two separate cohorts have been correlated with polymorphisms in the NLRP3 gene region in RA patients treated with anti-TNF-α. This is a good predictor of a true relationship between the gene and the response to treatment. The data also showed that polymorphisms of CARD8 rs2043211 were significantly associated with EULAR response to anti-TNF-α therapy. The significance of NLRP3-inflammasome in RA pathogenesis is demonstrated by the findings of increased mRNA NLRP3 in RA synovial tissue relative to osteoarthritis subjects [39]. In a cell expression analysis, Hitomi et al., 2009, found that rs4612666 variant allele to induce lower mRNA expression by reducing the transcriptional enhancer activity (in intron 7) of the NLRP3 gene [41]. However, because the association found in this polymorphism remained statistically important this SNP is functionally relevant for the outcome of anti-TNF-α treatment in RA patients [42].

The caspase-associated recruitment domain (CARD) is a conserved homology domain and a protein-protein interaction module [43], it is a member of the CARD protein family, has been proposed to be part of the inflammasome family proteins [44]. The inflammasome is a complex of cytoplasmic proteins that play a crucial role in the maturation and secretion of pro-inflammatory cytokines [45], such as interleukin (IL)-1β and IL-18 [46,47]. These cytokines are known to cause a range of infection-related biological effects, inflammation, and autoimmune processes and they are highly implicated in the pathogenesis of RA by activating the adaptive immune response [46,47] CARD8 has been shown to be a negative regulator of activation of NF-κB and caspase-1, suggest its role in suppressing NLRP3 inflammasome activation, this led to the immune response and inflammatory activity to be suppressed [44]. CARD8 is a new signaling molecule involved in the regulation of activation of caspase-1 and NF-κB [48] and plays a key role in the development of RA [47].

Genetic variants of CARD8 are also important in the pathogenesis of a variety of inflammatory diseases [49]. The gene polymorphisms of CARD8 have been associated with several inflammatory and immune diseases, such as RA [15,43], Crohn's disease [50], gout [51], ankylosing spondylitis [52], and atopic dermatitis [53]. A polymorphism of CARD8 and rs2043211, is associated with the risk of RA [43].

This study has some limitations. First, the sample size was relatively small because some patients discontinued treatment before 3 months, limiting the study power. Second, because autoimmune diseases are more common in women, we failed to recruit more men. RA patients almost over 40 years old and rarely have no medical issues or chronic diseases. Second, we investigated only two NLRP3 inflammasome polymorphisms, more SNPs should be assessed. Finally, because RA is genetically heterogeneous among ethnicities, our findings only apply to Iraqis.

## 5 Conclusions

The goal of this case-control study was to find out more about the association between RA and polymorphisms in the inflammasome-related genes (CARD8 and NLRP3) in Iraqi people. Neither etanercept nor infliximab is better than the other for treating RA. The study found an increase in the frequency of RA in older females, indicating that pathogenesis includes genetic and physiological differences. Both males and females had a positive response to anti-TNF-α therapy, but females had a poorer clinical response. No significant difference was found between the risk of RA and CARD8 rs2043211 and NLRP3 rs4612666 polymorphisms. However, we found that variant genotypes of CARD8 rs2043211 and NLRP3 rs4612666 were associated to a more active course of disease after 3 months of treatment, bad outcomes of disease activity, low response to anti-TNF-α therapy, and high levels of serum TNF-α and IL-1β.

## Author contribution statement

Ahmed Abdul-Hassan Abbas and Ahmed Sahib Abdulamir: Conceived and designed the experiments.

Abdullah Abbas Awni: Performed the experiments; Analyzed and interpreted the data; Contributed reagents, materials, analysis tools or data; Wrote the paper.

Zainab Oday Hamed: Analyzed and interpreted the data; Contributed reagents, materials, analysis tools or data.

## Data availability statement

Data included in article/supplementary material/referenced in article.

## Declaration of competing interest

The authors declare that they have no known competing financial interests or personal relationships that could have appeared to influence the work reported in this paper.
